# Persistent B Cell–Derived MHC Class II Signaling Is Required for the Optimal Maintenance of Tissue-Resident Helper T Cells

**DOI:** 10.4049/immunohorizons.2300093

**Published:** 2024-02-12

**Authors:** Young Min Son, In Su Cheon, Chaofan Li, Jie Sun

**Affiliations:** * Department of Systems Biotechnology, Chung-Ang University, Anseong, Republic of Korea; † Division of Pulmonary and Critical Care Medicine, Department of Medicine, Mayo Clinic College of Medicine and Science, Rochester, MN; ‡ Carter Immunology Center, University of Virginia, Charlottesville, VA;; § Division of Infectious Disease and International Health, Department of Medicine, University of Virginia, Charlottesville, VA

## Abstract

Emerging studies have identified the critical roles of tissue-resident memory CD8^+^ T (T_RM_) and B (B_RM_) cells in the protection against mucosal viral infections, but the underlying mechanisms regulating robust development of T_RM_ and B_RM_ cells remain incompletely understood. We have recently shown that tissue-resident helper CD4^+^ T (T_RH_) cells, developed following influenza virus infection, function to sustain the optimal maintenance of T_RM_ and B_RM_ cells at the mucosal surface. In this study, we have explored the cellular and molecular cues modulating lung T_RH_ persistence after influenza infection in C57BL/6 mice. We found that T_RH_ cells were colocalized in tertiary lymphoid structures (TLSs) with local B cells. Abolishing TLSs or the depletion of B cells impaired lung T_RH_ cell numbers. Of note, we found that persistent TCR signaling is needed for the maintenance of T_RH_ cells after the clearance of infectious influenza virus. Furthermore, selective ablation of B cell–derived MHC class II resulted in partial reduction of lung T_RH_ cell number after influenza infection. Our findings suggest that the interaction between lung-resident T_RH_ cells and B cells, along with persistent Ag stimulation, is required to maintain T_RH_ cells after respiratory viral infection.

## Introduction

A fundamental characteristic of the adaptive immune system is its capacity to establish immunological memory following the initial encounter with Ags. Upon secondary infection caused by the same virus or viruses harboring conserved T cell epitopes, memory T cells rapidly activate, undergo secondary effector T cell expansion and differentiation, and expeditiously mediate the clearance of pathogens before they can spread systemically. Besides the circulating memory T and B cells that patrol the whole body, studies in the past decade have also established the presence of mucosal-residing tissue-resident memory T (T_RM_) and B (B_RM_) cells, which provide immediate and superior protection against reinfection at the pathogen entry site (1, 2). For instance, we and others have previously reported the powerful effects of mucosal protection mediated by T_RM_ and B_RM_ cells against influenza viral infections (3–8). Notably, recent studies have also indicated that robust induction of mucosal T_RM_ and B_RM_ cells after mucosal vaccination may provide superior protection against SARS-CoV-2 reinfection (9–12).

Previously, we have found a population of lung PD1^hi^FR4^hi^ tissue-resident helper CD4^+^ T (T_RH_) cells following influenza virus infection and demonstrated that T_RH_ cells play a vital role in supporting the development and/or maintenance of CD8^+^ T_RM_ and B_RM_ cells in the respiratory tract (5). T_RH_ cells coexhibit features of follicular helper T cells and resident memory T cells, and their development is dependent on the transcription factors BCL6 and Bhlhe40 (5). Interestingly, T_RH_ cells are colocalized with B cells in lung tertiary lymphoid structures (TLSs) after influenza infection (5, 13). TLSs are ectopic lymphoid organs that develop in nonlymphoid tissues at sites of inflammation (14, 15). In the respiratory tract, certain types of TLSs have been reported as inducible bronchus-associated lymphoid tissue (iBALT), which can be detected in the lungs after exposure of pathogens, allergens, and harmful particulates (16, 17).

Given the important roles of T_RH_ cells in the development local CD8 and B cell immunity in the respiratory mucosa, it is critical to further dissect the underlying cellular and molecular mechanisms regulating T_RH_ development and/or maintenance. Previously it was shown that B cells and persistent MHC class II (MHCII) signaling are critical for T_RH_ maintenance (13), but the cell types that provide the persistent MHCII signaling to sustain T_RH_ cells are currently unknown. Furthermore, despite their localization in iBALT, the roles of iBALT in maintaining T_RH_ cell development and phenotypes are unknown. In this study, we have gone on to examine the roles of Ag persistence, B cells, and TLSs in regulating the development and maintenance of T_RH_ cells after influenza infection. We found that T_RH_ cells were colocalized mainly in iBALT with B cells, and abolishing TLSs or the depletion of B cells impaired lung T_RH_ cell persistence. Furthermore, we found that persistent TCR signaling was needed for the maintenance of T_RH_ cells, and selective ablation of B cell–derived MHCII caused partial reduction of lung T_RH_ levels after influenza infection. Our findings have revealed new insights into the regulation of T_RH_ cells in respiratory traction after viral infection.

## Materials and Methods

### Mice and influenza viral infection

Wild-type (WT) C57BL/6, CD45.1, μMT, and IL-21 VFP reporter mice were purchased from The Jackson Laboratory and bred in-house. To generate CD45.1^+^ and CD45.2^+^ (CD45.1^+^/2^+^) mice, CD45.1^+^ mice were crossed with C57BL/6 mice. CD45.1^+^ mice were crossed with OTII mice, and OTII F_1_ mice were additionally crossed with CD45.1^+^ mice to generate CD45.1^+^ OTII mice. We crossed MHCII^fl/fl^ mice with Ubc^CreERT2^ transgenic mice to generate MHCII^fl/fl^Ubc^CreERT2^ mice. All animal protocols were approved by the Institutional Animal Care and Use Committees of the Mayo Clinic (Rochester, MN). Mice of both sexes aged 8–10 wk were used in the experiments. The mice were intranasally infected under anesthesia with influenza A/PR8/34 (PR8) at a dose of ∼200 PFU per mouse, as described previously (18). In some experiments, CD45.1^+^ OTII cells were adoptively transferred into CD45.1^+^/2^+^ congenic mice. After 1 d, PR8-OTII (∼5.0 × 10^3^ PFU/mouse) (19) was infected intranasally.

### Intravascular labeling with anti-CD45 and preparation of lung cell suspension

Mice were i.v. injected with 2 μg of anti-CD45 (clone 30-F11; Tonbo Biosciences), which was diluted in 300 μl of sterile PBS 5 min before sacrificing the mice. To prepare single cells from the lung tissue, the lung was cut into small pieces, digested with type 2 collagenase (Worthington Biochemical), and dissociated at 37°C for 30 min using gentleMACS (Miltenyi Biotec). The cells were further processed through a 70-μm cell strainer (Falcon) and washed with plain IMDM (Gibco). After red blood cell lysis, the cells were centrifuged and resuspended in cold FACS buffer (PBS, 2 mM EDTA, 2% FBS, and 0.09% sodium azide) for flow cytometry analysis. Lung circulating immune cells are i.v. Ab^+^, and lung tissue immune cells are defined as i.v. Ab^−^.

### Ab administration in vivo

Influenza-infected WT mice were treated with control IgG or various neutralizing or depleting Abs as described in the corresponding results sections. Lymphotoxin-β receptor Ig fusion (LTβR-Ig; 250 μg) obtained from Dr. Yangxin Fu’s laboratory (20) was administered for TLS elimination, and 500 μg of CD20 Ab (clone 5D2, Genentech) was administered for B cell depletion by i.p. injection at 14 and 21 days postinfection (dpi). For neutralizing MHCII signaling, mouse MHCII (clone M5/114, Bio X Cell) Ab was injected into WT mice. The first dose was 1 mg at 14 dpi, and the second dose was 0.5 mg at 21 dpi FTY720 (1 mg/kg; Cayman Chemical) administrated via i.p. injection daily from 13 dpi onward to block lymphocyte migration until the mice were sacrificed (5).

### Tamoxifen treatment

To induce gene recombination in MHCII^fl/fl^Ubc^CreERT2^ mice, tamoxifen (Sigma-Aldrich) was diluted in warm sunflower oil (Sigma-Aldrich) and daily administered (2 mg per mouse) via the i.p. route for 5 consecutive days.

### Immunofluorescence

The left lobe of the whole lung was harvested and fixed in 4% paraformaldehyde solution overnight at 4°C. The fixed sample was sequentially incubated in 15 and 30% sucrose solutions in PBS for 12 h each. Subsequently, the sample was embedded with OCT compound (Sakura Finetek) and stored at –80°C. For Ab staining and immunofluorescence imaging, lung sections were blocked with SuperBlock blocking buffer (Thermo Fisher Scientific) for 1 h at room temperature. B220 eFluor 660 (clone 4SM95, Invitrogen), CD4 eFluor 570 (clone RA3-6B2, Invitrogen), and/or GL7 Alexa Fluor 488 (clone GL7, BioLegend) Abs were stained on the lung tissue sections overnight at 4°C. After washing in 0.1% PBST (PBS with Tween 20), the slides were counterstained with DAPI and mounted. Tissue staining was observed, and representative images were captured using a Zeiss LSM 780 confocal system (Carl Zeiss).

### B cell Ags

The influenza PR8-hemagglutinin (HA) protein was a gift from M.C. Crank (National Institutes of Health). PR8-nucleoprotein (NP) was purchased from Sino Biological. Purified Ags were biotinylated using an EZ-Link sulfo-NHS-LC biotinylation kit (Thermo Fisher Scientific) with a biotin-to-Ag ratio of 1:1.3 M. To create tetramers, biotinylated Ags were combined with streptavidin-PE (PJ27S; ProZyme) at the predetermined ratio or a 5:1 ratio based on the biotin concentration provided by the manufacturer, as described previously (21). After a 30-min incubation on ice, any unconjugated biotinylated Ag was removed by several rounds of dilution and concentration using a 100-kDa Amicon ultra (MilliporeSigma) or 300-kDa Nanosep centrifugal devices (Pall). The tetramers were stored at 1 μM in 1× Dulbecco’s PBS at 4°C before use.

### Flow cytometry analysis

Cells were incubated with the appropriate Ab cocktail in FACS buffer for 30 min at 4°C in dark. The I-A^b^ NP_311–325_ tetramer, which was obtained from the National Institutes of Health Tetramer Core Facility, was used for primary staining of cells for 1 h at room temperature before other Ab surface staining. Then, the cells were washed with FACS buffer. FACS Abs were primarily purchased from BioLegend, eBioscience, or Tonbo Biosciences. The clone numbers of these Abs were as follows: CD45 (clone 30-F11), CD45.1 (clone A20), CD45.2 (clone 104), CD4 (clone RM4-5), CD44 (clone IM7), PD-1 (clone 29F.1A12), FR4 (clone eBio12A5), GITR (clone DTA-1), B220 (clone RA3-6B2), GL7 (clone GL7), CD38 (clone 90), IgD (clone 11-26c.2a), IgM (clone 11/41), CXCR5 (clone SPRCL5), and streptavidin-allophycocyanin. After Ab staining, the cells were analyzed using an Attune NxT system (Life Technologies). Data analysis was performed using FlowJo software (Tree Star).

### Mixed bone marrow chimera generation

For the generation of mixed bone marrow chimeric mice, CD45.1 recipient mice were lethally irradiated (1100 rad). Mixed donor cells were prepared by mixing 1 × 10^6^ bone marrow cells from MHCII^fl/fl^Ubc^CreERT2^ or WT mice with 4 × 10^6^ bone marrow cells from μMT mice. These donor cells were i.v. injected into the irradiated CD45.1 recipient mice. Experimental chimeric mice were infected with PR8 at 8 wk after reconstitution.

### Quantitative RT-PCR

To measure the expression levels of *Il21*, *Bcl6*, *Cxcr5*, and *Foxp3*, WT or IL-21 VFP reporter mice were infected with influenza PR8 for 28 d. Then, the indicated cells were sorted out from CD45_i.v._^−^CD4^+^ tissue-resident T cells from infected mice using a FACSAria (BD Biosciences). Total RNA was extracted from the sorted cells using a total RNA purification kit (Sigma-Aldrich) and treated with DNase I (Invitrogen). Random primers (Invitrogen) and Moloney murine leukemia virus reverse transcriptase (Invitrogen) were used to synthesize first-strand cDNAs. Quantitative PCR and data analysis were performed as described previously (22). Hypoxanthine phosphoribosyltransferase (HPRT) was used as the housekeeping gene control. The following primers were used for amplification: *Hprt*, forward, 5′-CTCCGCCGGCTTCCTCCTCA-3′, reverse, 5′-ACCTGGTTCATCATCGCTAATC-3′; *Il21*, forward, 5′-CGCTCACGAATGCAGGAGTA-3′, reverse, 5′-GTCTGTGCAGGGAACCACAA-3′; *Bcl6*, forward, 5′-CCGGCTCAATAATCTCGTGAA-3′, reverse, 5′-GGTGCATGTAGAGTGGTGAGTGA-3′; *Cxcr5*, forward, 5′-TGGCCTTCTACAGTAACAGCA-3′, reverse, 5′-GCATGAATACCGCCTTAAAGGAC-3′; *Foxp3*, forward, 5′-CAC CCA GGA AAG ACA GCA ACC-3′, reverse, 5′-GCA AGA GCT CTT GTC CAT TGA-3′.

### Statistical analysis

Graphs were generated using the GraphPad Prism software. Statistical significance was evaluated by calculating *p* values using a paired or unpaired Student *t* test (two-tailed). Differences with *p* values <0.05 were considered statistically significant.

## Results

### TLS is required for IL-21^hi^CD4^+^ T_RH_ cell maintenance

**FIGURE 1.**
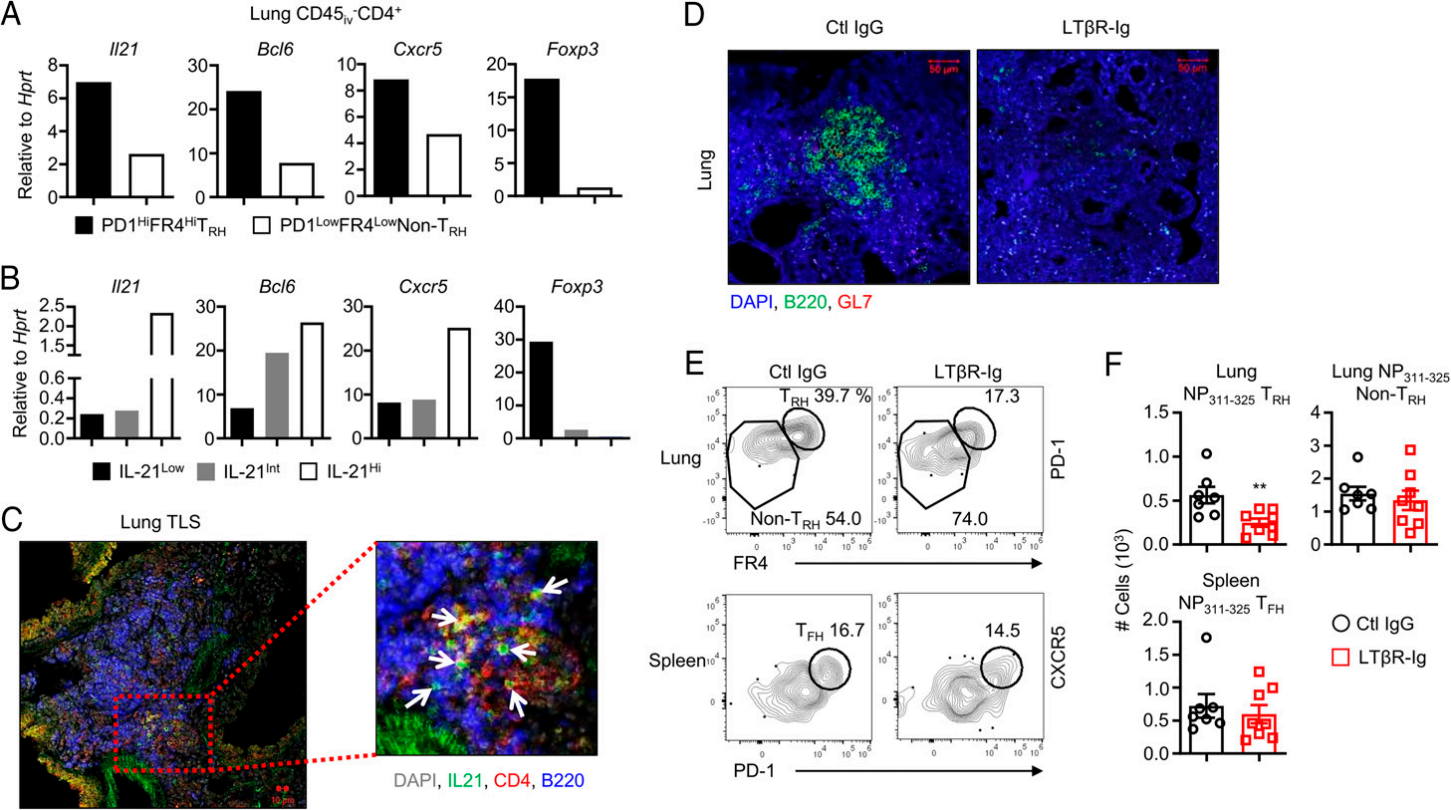
Features and localization of IL-21^+^ T_RH_ cells. (**A**) *IL21*, *Bcl6*, *Cxcr5*, and *Foxp3* were measured by quantitative RT-PCR in the T_RH_ (CD45_i.v._^−^CD4^+^CD44^+^PD1^hi^FR4^hi^) or non-T_RH_ (CD45_i.v._^−^CD4^+^CD44^+^PD1^lo^FR4^lo^) cells sorted from influenza-infected mice (pooling lung cells of 12 mice). (**B**) Expression of the indicated genes was examined in IL-21^hI^, IL-21^int^ or IL-21^lo^ cells that were sorted from lung CD45_i.v._^−^CD4^+^CD44^+^GITR^−^ cells of influenza-infected mice (pooling cells of 10 mice). (**C**) Lung IL-21^+^CD4^+^ T cells were detected with B cells in TLSs from influenza-infected IL-21 VFP mice (green, IL-21 VFP; red, CD4; blue, B220). The representative image is from at least two independent experiments. (**D**–**F**) Control IgG or LTβR-Ig was i.p. injected into C57BL/6 WT mice at 14 and 21 dpi, after which the number of lung NP-specific T_RH_, non-T_RH_, and spleen T_FH_ cells were observed. The representative image (D) and dot plot (E) from at least two independent experiments (three to four mice per group) are shown. Scale bars, 10 μm (C), 50 μm (D). All experiments were conducted at 28 dpi. Statistical analysis was performed with an unpaired Student *t* test. **p < 0.01.

We have previously demonstrated that CD45_i.v._^−^CD4^+^CD44^+^PD1^hi^FR4^hi^ T_RH_ cells expressed BCL6 and high levels of IL-21 following influenza virus infection (5). Furthermore, BCL6^+^ T_RH_ cells have been identified inside TLSs, whereas T-bet^+^ T_H1_ cells were located outside of this structure at 30–60 dpi (13). However, whether TLS is required for T_RH_ cell maintenance is not clear. To address the question, we first assessed the characteristics of T_RH_ cells following influenza virus infection. Lung total T_RH_ and non-T_RH_ (CD45_i.v._^−^CD4^+^CD44^+^PD1^lo^FR4^lo^) cells were sorted (Supplement Fig. 1A), and the gene expression levels of *Il21*, *Bcl6*, *Cxcr5*, and *Foxp3* were measured by quantitative RT-PCR. In line with previous studies (5, 13), *Il21*, *Bcl6*, and *Cxcr5* were highly expressed in T_RH_ cells, but these cells also expressed higher levels of *Foxp3*, compared to non–T_RH_ cells, potentially due to contamination of small percentages of regulatory T cells in this gating strategy (Fig. 1A). Next, we sorted IL-21^lo^, IL-21^int^, and IL-21^hi^ cells (Supplement Fig. 1B) from CD45_i.v._^−^CD4^+^GITR^−^CD44^+^ lung cells from influenza-infected IL-21-VFP reporter mice (at 28 dpi). We found that IL-21^hi^ cells exhibited the highest expression levels of *Il21*, *Bcl6*, and *Cxcr5* but not *Foxp3*, compared with other cell populations (Fig. 1B), suggesting that T_RH_ cells but not regulatory T cells express IL-21 in the lung after influenza infection.

To assess the colocalization of IL-21^+^ T_RH_ and local B cells, we conducted confocal microscope imaging. Our observations revealed that most IL-21^+^ CD4^+^ T cells were located near B cells within the TLS (Fig. 1C). This led us to investigate the requirement of a TLS for the maintenance of T_RH_ cells after influenza infection. To this end, we eliminated the TLS using LTβR-Ig treatment, which is known to be able to destroy TLSs in various models (23–25). We administered LTβR-Ig at 14 and 21 dpi and observed a clear removal of TLSs in the lung at 28 dpi compared to the IgG-treated control group (Fig. 1D). Notably, influenza nucleoprotein epitope (NP_311–325_)–specific T_RH_ cells were significantly decreased in the LTβR-Ig–treated group compared to the control group, while Ag-specific non–T_RH_ cells and spleen follicular helper T (T_FH_) cells showed no significant differences between the two groups (Fig. 1E–F). In our previous study, we showed that T_RH_ cells supported the formation of germinal center (GC) B cells (GL7^+^CD38^−^) and HA-specific tissue-resident memory B cells (B_RM_: IgD^−^IgM^−^CD38^+^HA^+^), while aiding the maintenance of CD8^+^ T_RM_ cells. Correspondingly, decreased T_RH_ cells resulted in impaired adaptive immunity, rendering the host significantly vulnerable to heterologous influenza reinfection (5). Therefore, we examined tissue B cell persistence and found a significant decrease of lung-resident B (CD45_i.v._^−^B220^+^), GC B, and HA^+^ B_RM_ cells in lungs from LTβR-Ig–treated mice (Supplement Fig. 2). These results suggest that TLS contributes to the maintenance of Ag-specific T_RH_ cells following influenza virus clearance.

### Tissue-resident B cells are involved in the maintenance of T_RH_ cells

**FIGURE 2.**
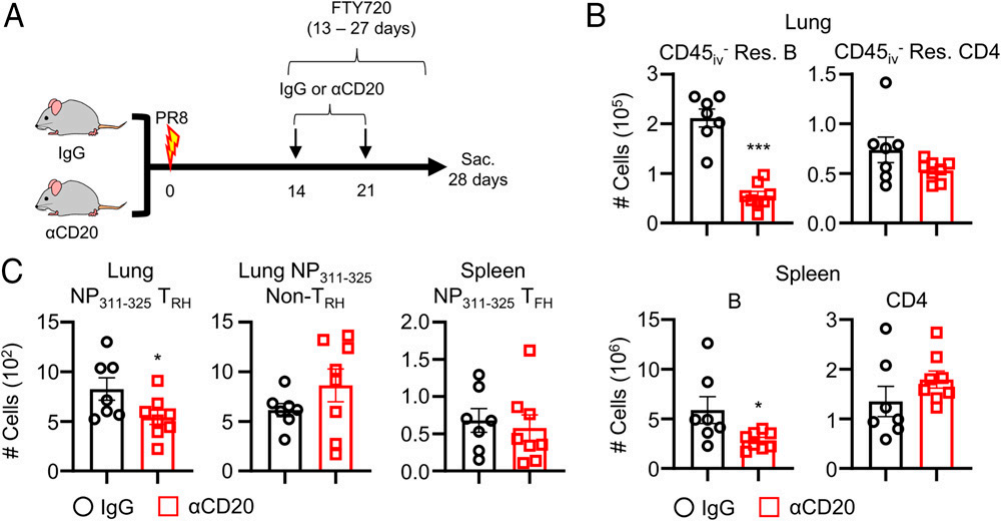
Tissue-resident B cells are required for the maintenance of T_RH_ cells. WT mice were infected with influenza. (**A**) WT mice were treated with anti-CD20 or IgG at 14 and 21 dpi and FTY720 was administered daily from 13 dpi onward. (**B**) Top, Lung B and CD4^+^ T cells. Bottom, Spleen B and CD4^+^ T cells. (**C**) Lung NP-specific T_RH_, non-T_RH_, and spleen T_FH_ cells were measured by flow cytometry. The mice were sacrificed at 28 dpi. Pooled results from two independent experiments (each group *n* = 3–4) are shown. Statistical analysis was performed with an unpaired Student *t* test. *p < 0.05, ***p < 0.001.

The diminished T_RH_ cell numbers following TLS ablation led us to examine whether lung B cells are required for the maintenance of T_RH_ cells. To this end, we conducted B cell depletion after influenza infection. To exclude the potential effects of circulating B cells in affecting T_RH_ cells, we treated the mice with anti-CD20 in the presence of daily injection of FTY720, which prevents lymphocyte egress from lymphoid tissues (5), starting from 13 dpi (Fig. 2A). Treatment with anti-CD20 dramatically depleted both lung-resident B and spleen B cells, while not affecting the lung and spleen total CD4^+^ T cell population (Fig. 2B). However, B cell depletion in the lung led to a significant decrease in the number of lung NP-specific T_RH_ cells compared to the control group, whereas no significant differences in the lung NP-specific non-T_RH_ and spleen T_FH_ were observed between the IgG and anti-CD20 groups (Fig. 2C). These results indicate that local tissue-resident B cells are involved in the maintenance of Ag-specific T_RH_ cells.

### Persistent A stimulation is required to maintain T_RH_ cells in the lung

Infectious influenza virus is usually cleared in the respiratory tract by the immune system within 10 dpi (26, 27), but influenza Ag, particularly the NP protein, appears to be persistent for a couple of months after infection. Because prior studies have suggested that persistent MHCII signaling may be required for the maintenance of T_RH_ cells (13), we explored whether persistent TCR signaling is required for the generation or maintenance of T_RH_ cells.

**FIGURE 3.**
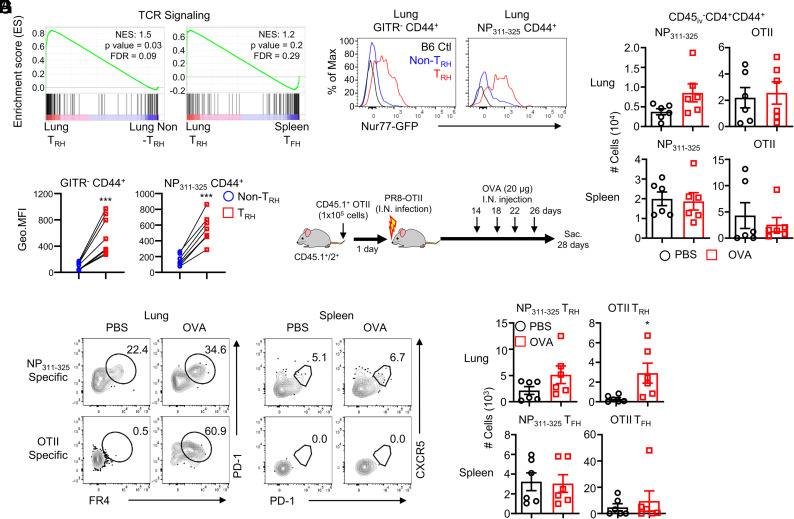
Persistent Ag stimulation is required for T_RH_ cell maintenance. (**A**) Enrichment of TCR signaling-related molecule expression between (left) lung T_RH_ versus lung non–T_RH_ cells and (right) lung T_RH_ versus spleen T_FH_ cells. Original bulk RNA sequencing data were extracted from GSE153226. (**B** and **C**) Expression of Nur77 was measured by flow cytometry from influenza-infected Nur77 GFP mice at 28 dpi. Pooled results from three independent experiments (each group *n* = 2–3) are shown. (**D**) CD45.1^+^ OTII cells were transferred into CD45.1^+^/2^+^ host mice. One day later, mice were intranasally infected with recombinant PR8 virus with an OTII epitope (PR8-OTII). Mice were treated with OVA or PBS every 4 d from day 14 to day 26. The mice were then sacrificed at 28 dpi. (**E**) NP-specific or OTII-specific CD4^+^ T cells in the lung (top) or spleen (bottom) were measured. (**F** and **G**) NP-specific or OTII-specific cells were analyzed in the lung (top, T_RH_) and spleen (bottom, T_FH_). Pooled results from two independent experiments (each group *n* = 2–4) are shown. Statistical analysis was performed with a paired (C) or unpaired (G) Student t test. *p < 0.05, ***p < 0.001.

Reanalysis of our previously published bulk RNA sequence data (Gene Expression Omnibus: https://www.ncbi.nlm.nih.gov/geo/, GSE153226) (5) found that lung T_RH_ cells were enriched with TCR signaling-related genes compared to lung non-T_RH_ or spleen T_FH_ cells (Fig. 3A). To further investigate whether T_RH_ cells continuously receive TCR signaling after viral clearance, we infected Nur77-GFP mice with influenza virus and measured Nur77-GFP expression levels. Nur77 is a downstream signaling molecule activated following TCR stimulation (3), and its expression was widely used as a surrogate of TCR signaling (3, 28). Both lung total T_RH_ and NP-specific T_RH_ cells exhibited significantly higher levels of Nur77-GFP expression compared to non–T_RH_ cells (Fig. 3B, 3C). These results support the hypothesis that lung T_RH_ cells exhibit ongoing TCR stimulation.

To explore this hypothesis further, we conducted repeated Ag stimulation test and measured the endogenous NP-specific T_RH_ cells and transferred OTII-specific T_RH_ cells (Supplement Fig. 3). CD45.1^+^ OTII cells were transferred into CD45.1^+^/2^+^ host mice, and the mice were infected with PR8 virus expressing the chicken OVA_323–339_ (OTII) peptide (PR8-OTII) (19) 1 d later. OVA or PBS was intranasally injected every 4 d from 14 to 26 dpi (Fig. 3D). The total numbers of lung-resident endogenous NP-specific and transferred OTII-specific CD4^+^ T cells have shown no differences between the OVA- or PBS-treated group in either the lung or spleen (Fig. 3E). The number of endogenous NP-specific lung T_RH_ cells and spleen T_FH_ cells also showed no differences between the PBS- and OVA-treated groups. Interestingly, OTII-specific lung T_RH_ cells were not detected in the PBS injection group likely due to the lack of Ag persistence, as the OTII peptide was inserted to the less abundant H1 protein locus rather than the NP locus (which is a more abundant protein during infection) (19, 29, 30). Nevertheless, repeated OVA treatment in the lung fostered T_RH_ development within OTII cells (Fig 3F–G), suggesting that persistent Ag stimulation promotes T_RH_ development after respiratory viral infection.

### Persistent local MHCII signaling is required to maintain Ag-specific T_RH_ cells following virus infection

**FIGURE 4.**
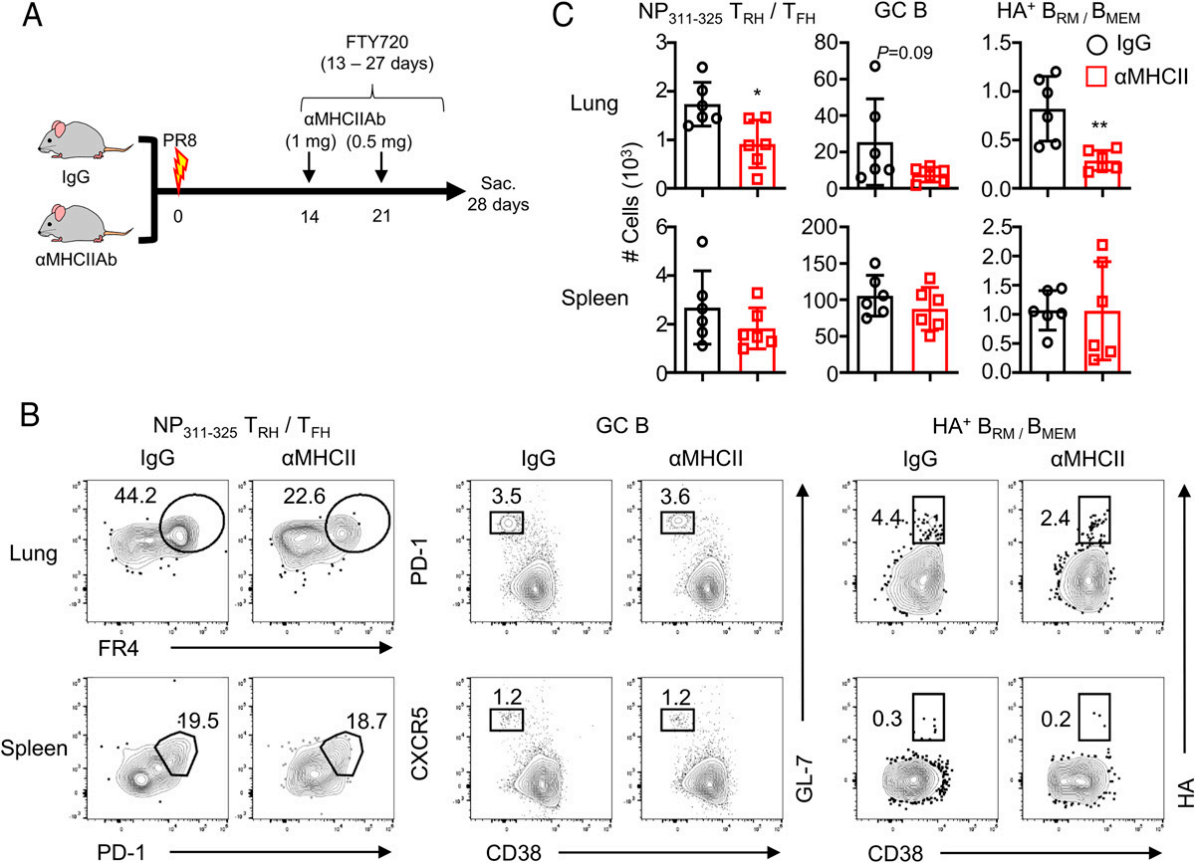
Requirement of local MHCII signaling for maintenance of lung Ag-specific T_RH_ cells. WT mice were infected with influenza PR8. (**A**) WT mice were injected with anti-MHCII or IgG at 14 and 21 dpi together with FTY720 treatment. The mice were sacrificed at 28 dpi. (**B** and **C**) NP-specific lung T_RH_ or spleen T_FH_ cells, lung/spleen GC B cells, or HA^+^ B_RM_/memory B (B_MEM_) cells were measured by flow cytometry. Pooled results from two independent experiments (each group *n* = 2–4) are shown. Statistical analysis was performed with an unpaired Student *t* test. *p < 0.05, **p < 0.01.

Ag-loaded MHCII is the major signal to stimulate CD4^+^ T cells, and the deficiency of MHCII signaling reduced the number of T_RH_ cells in the lung (13). However, whether lung-resident MHCII signaling is required for T_RH_ cell maintenance is unclear. Therefore, to examine the roles of local MHCII signaling in sustaining lung Ag-specific T_RH_ cells, we blocked the MHCII signaling at 14 and 21 dpi together with FTY720 treatment (Fig. 4A). The numbers of lung NP-specific T_RH_ cells and HA^+^ B_RM_ cells were significantly reduced in the group with MHCII signaling blockade compared to those of the control group (Fig. 4B, 4C). These findings indicate that local MHCII signaling contributes to the formation of lung Ag-specific T_RH_ and B_RM_ cells.

### MHCII signaling from B cells is required for the optimal maintenance of Ag-specific T_RH_ cells

**FIGURE 5.**
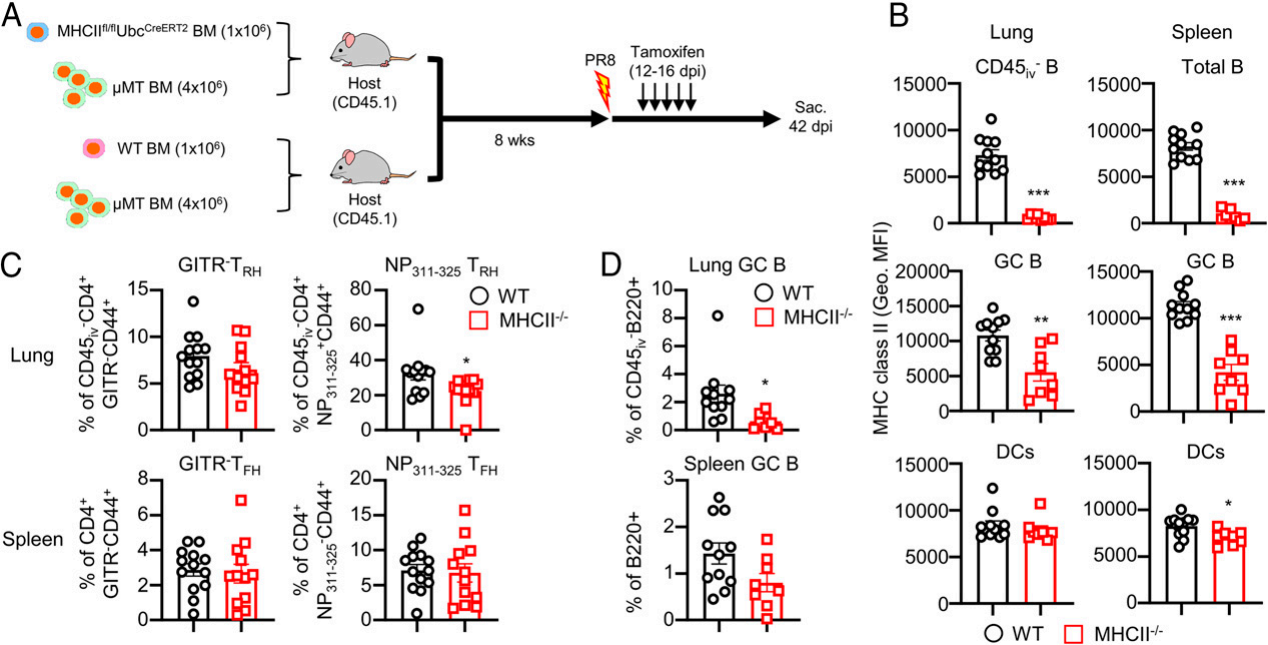
Effect of MHCII signaling from local B cells to maintain lung T_RH_ cells. (**A**) Mixed bone marrow chimera mice for generating inducible MHCII deficiency in B cells. After bone marrow reconstitution, mice were infected with influenza PR8, after which tamoxifen was injected daily from 12 to 16 dpi to deplete MHCII. (**B**) Expression of MHCII was detected in total B, GC B, or DCs from lung and spleen. (**C**) Lung GITR^−^ T_RH_ or NP-specific T_RH_ cells (top) and spleen GITR^−^ T_FH_ or NP-specific T_FH_ cells (bottom) were measured. (**D**) Frequencies of lung or spleen GC B cells were measured. (B and D) Pooled results from three independent experiments (each group *n* = 2–4). (C) Pooled results from four independent experiments (each group *n* = 2–4). All experiments were conducted at 42 dpi. Statistical analysis was performed with an unpaired Student *t* test. *p < 0.05, **p < 0.01, ***p < 0.001.

To specifically investigate the role of the MHCII signal from B cells in the maintenance of Ag-specific T_RH_ cells, we generated a mixed bone marrow chimera mouse model, in which we can specifically deplete MHCII in B cells in an inducible fashion (Fig. 5A). Lethally irradiated mice were reconstituted with bone marrow cells from μMT mice (lacking B cells [31]) mixed with MHCII^fl/fl^Ubc^CreERT2^ (termed the MHCII^−/−^ group) or WT mice bone marrow (WT group) at 4:1 ratio. After 8 wk of reconstitution, the mice were infected with influenza virus. Tamoxifen was administered consecutively for 5 d at 12–16 dpi to deplete MHCII in B cells (Fig. 5A). The expression of MHCII was dramatically reduced at lung-resident B, lung GC B, spleen B, and spleen GC B cells in the MHCII^−/−^ group, whereas dendritic cells (DCs) still expressed comparable levels of MHCII between the WT and MHCII^−/−^ groups (Fig. 5B), confirming the successful achievement of B cell–specific MHCII deficiency. In this animal model, we found that lung GC B cells were decreased in the MHCII^−/−^ group compared to the WT group, and lung NP-specific T_RH_ cells were moderately affected after MHCII ablation in B cells (Fig. 5C, 5D). Thus, we conclude that the local MHCII signal presented by B cells partially contributes to the maintenance of local Ag-specific T_RH_ cells at later time points.

## Discussion

T_RH_ cells are essential for local adaptive immunity, especially after respiratory viral infections. They help maintain T_RM_ cells after viral clearance and support local GC B and optimal B_RM_ cells. Consistent with this notion, there is evidence of IL-21 from local CD4^+^ T cells influencing the development of T_RM_ cells in the brain (32). Interestingly, we found IL-21 VFP^+^ T_RH_ cells interacting with B cells in the TLS at 28 days after influenza infection. The importance of TLS in the respiratory tract, especially the iBALT generated after viral infection, has been emphasized recently (17). We noted a marked reduction in T_RH_ cell numbers when TLS was disrupted, suggesting that T_RH_ cell development and/or maintenance require the support of local environment at the TLS.

Persistent TCR-MHC signaling has been suggested to impact the formation of T_RM_ cells. Blocking MHCI or lacking Nurr77 results in fewer T_RM_ cells after influenza infection (3). Furthermore, continuous TCR signaling aids T_RM_ cell tissue migration while inhibiting their exit to blood (33). Analyzing RNA sequencing data, we observed that T_RH_ cells displayed high levels of TCR signaling molecules such as Nur77, hinting at the necessity of persistent Ag stimulation for T_RH_ cell maintenance. We further delved into the cellular mechanisms that uphold T_RH_ cells within the TLS after viral infections and found that B cell MHCII was required for the optimal maintenance of T_RH_ cells. This is not particularly surprising given that B cell Ag presentation is needed for the generation and maintenance of T_FH_ cells in the secondary lymphoid organ. Notably, B cell MHCII deficiency only partially impaired T_RH_ cell maintenance, while the blockade of MHCII signaling from all cell types greatly abrogated T_RH_ cell preservation (13, 34, 35). These data suggest that persistent Ag presentation by other cells is likely also involved in the maintenance of T_RH_ cells. Studies have pinpointed CD11c^hi^ DCs in TLS formation and lung epithelial cells in forming T_RM_ cells (16, 36). Therefore, it is possible that DCs or the epithelial cell–derived Ag/MHCII complex may also be involved in maintaining T_RH_ cells. Such possibilities require future investigations.

In conclusion, in this study, we found that sustaining T_RH_ cells after local viral clearance relies on the presence of TLS and continuous Ag stimulation. Given the importance of mucosal immunity in rapid constraining respiratory viral dissemination and the emerging roles of T_RH_ cells in regulating mucosal immune cells, we propose that innovative immunization strategies, offering TLS biogenesis and prolonged local Ag release, may be the key for the success of mucosal vaccines against respiratory viral infections through the bolstering of local T_RH_ and memory B and CD8^+^ T cells.

### Limitations of the study

Although we used OTII peptide administration to induce persistent TCR signaling in OTII cells after PR8-OTII infection, we have not confirmed whether persistent TCR signaling was indeed induced in OTII cells and, if so, how long the signaling lasted. Further studies using the transfer Nur77-GFP OTII cells to report TCR signaling in vivo after OTII peptide inoculation could address this limitation. Additionally, B cells are the major cell types forming TLSs in the respiratory tract, and B cell depletion with anti-CD20 treatment is expected to impair TLS formation (PMID: 28355561). Thus, it is possible that the diminished T_RH_ cells after anti-CD20 treatment are due to the absence of TLS, rather than the effects of the absence of direct B–T cell communication (such as Ag presentation). This possibility necessitates further studies in the future.

## Supplementary Material

Supplemental Figures 1 (PDF)
