# Supplementary material for: Persistent B Cell–Derived MHC Class II Signaling Is Required for the Optimal Maintenance of Tissue-Resident Helper T Cells
Source: Immunohorizons. 2024 Feb 12;8(2):163–71. doi: 10.4049/immunohorizons.2300093 (PMC10916357; doi:10.4049/immunohorizons.2300093)
Supplement: Supplemental Figures 1 (PDF) [file IH_2300093_Supplemental_1.pdf]

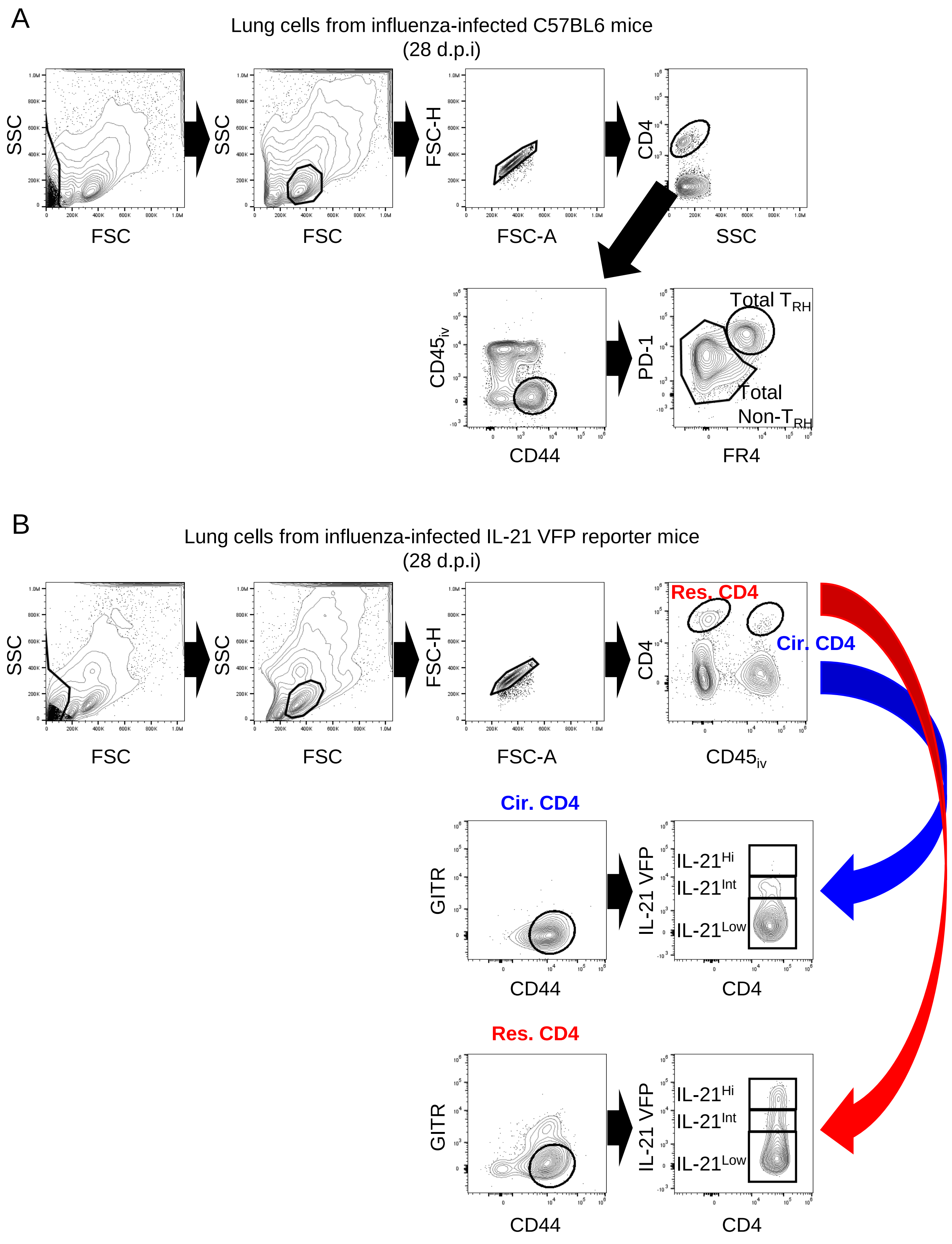

Supplement Figure 1. Gating strategies. A. Gating strategy to isolate total lung T_{RH} ($CD45_{i.v.}^{-}CD4^{+}CD44^{+}PD1^{Hi}FR4^{Hi}$) or non-$T_{RH}$ ($CD45_{i.v.}^{-}CD4^{+}CD44^{+}PD1^{Low}FR4^{Low}$) cells from influenza-infected mice (pooling lung cells of 12 mice). B. Gating strategy to sort IL-21^{Hi}, $^{Intermediate\ (Int)}$ or ^{Low} cells from lung $CD45_{i.v.}^{-}CD4^{+}CD44^{+}GITR^{-}$ cells of influenza-infected mice (pooling cells of 10 mice).

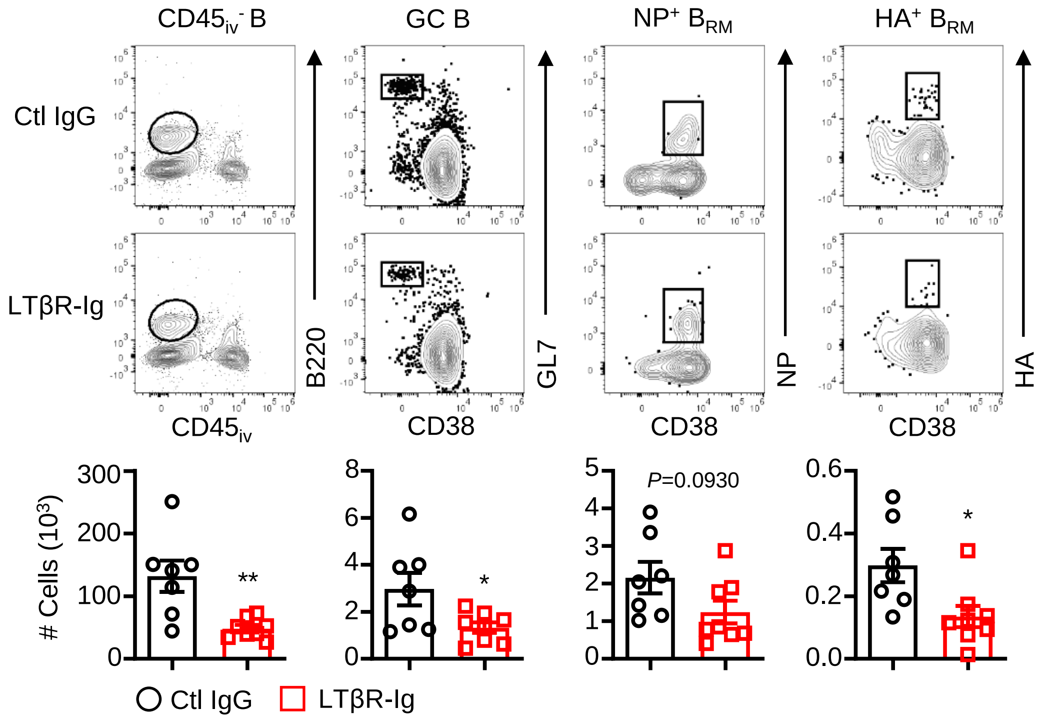

Supplement Figure 2. Impairment of lung B cell numbers after TLS ablation. Lung resident B, GC B, NP or HA-specific BRM cells were measured in Ctl IgG or LTβR-Ig treated groups. Ctl IgG or LTβR-Ig was intraperitoneal injected into WT mice at 14 and 21 d.p.i. Pooled results from at least two independent experiments (three to four mice per group). Statistical analysis was performed with unpaired student's t-test. *P<0.05.

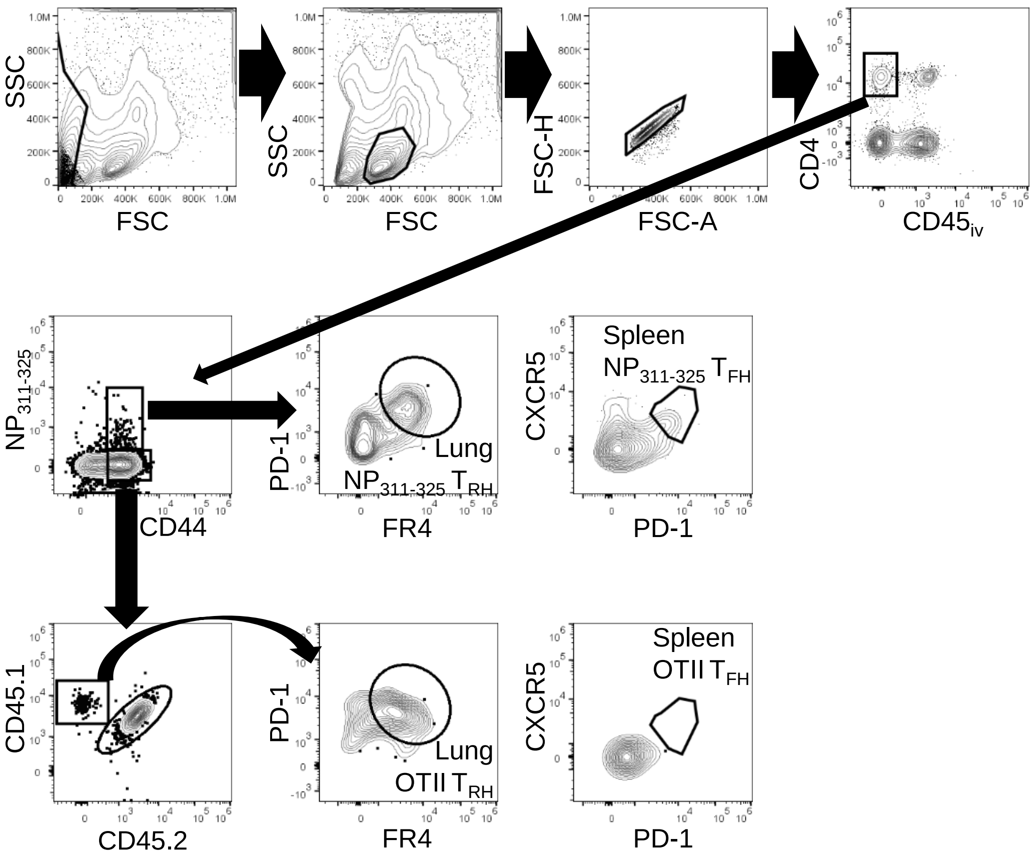

Supplement Figure 3. Gating strategies distinguishing $NP_{311\text{-}325}$ or OVA specific T_{RH} cells. All cells excluding debris were additionally gated for lymphocytes, then singlet cells were gated. Resident $CD45_{i.v.}^{-}$ $CD4^{+}$ T cells were gated and $NP_{311\text{-}325}^{+}CD44^{+}$ cells were analyzed to gate lung NP^{+} T_{RH} or spleen T_{FH} cells. The $NP_{311\text{-}325}^{-}$ $CD44^{+}$ cells were additionally gate to $CD45.1^{+}$ cells then lung $OTII^{+}$ T_{RH} or spleen T_{FH} cells were measured.
